# Diffuse large B-cell and follicular lymphoma presenting as a slowly growing compressive goiter: A case report and literature review

**DOI:** 10.1016/j.ijscr.2020.06.029

**Published:** 2020-06-27

**Authors:** Nicole Lin, Susana Vargas-Pinto, Savanah Gisriel, Mina Xu, Courtney E. Gibson

**Affiliations:** a University of Miami, Miller School of Medicine, Miami, FL, United States; b Section of Endocrine Surgery, Department of Surgery, Yale University School of Medicine, New Haven, CT, United States; c Department of Pathology, Yale University School of Medicine, New Haven, CT, United States

**Keywords:** TL, thyroid lymphoma, FNAC, fine needle aspiration cytology, DLBCL, diffuse large B cell lymphoma, FL, follicular lymphoma, IHC, immunohistochemistry, MZL, marginal zone lymphoma, FDG-18 PET, fluodeoxyglucose-18 positron emission tomography, ACR-TIRADS, American College of Radiologists Thyroid Imaging Reporting and Data Systems, Thyroid lymphoma, Goiter, Thyroid lobectomy, Diffuse large B cell lymphoma, Case report

## Abstract

**Introduction:**

Neck ultrasonography with fine-needle aspiration cytology (FNAC) is the diagnostic modality of choice for clinicians who routinely work up a thyroid mass. Distinguishing chronic lymphocytic infiltration from a lymphoproliferative process with FNAC in patients with Hashimoto’s thyroiditis presenting with a goiter can be particularly challenging.

**Case description:**

A 58 y.o. female with a history of a goiter showing interval growth and compressive symptoms over 18 months, was treated with a thyroid lobectomy. Surgical pathology demonstrated a thyroid lymphoma (TL) with mixed follicular and diffuse large B cell (DLBCL) components, not initially diagnosed by FNAC. Staging workup showed the involvement of chest lymph nodes only, consistent with Stage IIE disease. She was treated with combination chemotherapy and immunotherapy, followed by involved-field radiotherapy.

**Discussion:**

TL often arises in a background of chronic lymphocytic thyroiditis which can make its histological diagnosis a challenge. The disease is heterogeneous in histological subtype and progression.

**Conclusion:**

While TL usually presents as a rapidly growing neck mass, indolent types can present as a slow growing mass with subsequent transformation. Patients may benefit from avoiding unnecessary diagnostic steps, including surgery, and potential delays in treatment by performing a core needle biopsy when a lymphoproliferative process cannot be excluded if FNAC was initially performed.

## Highlights

• Rapid, but not slowly growing goiters are generally concerning for malignancy.
• Lymphoma represents < 5% of all primary thyroid malignancies.
• Hashimoto’s thyroiditis is a known risk factor for thyroid lymphoma.
• Core needle biopsy is highly sensitive for diagnosis of thyroid lymphoma.
• Multimodal treatment of thyroid lymphoma is preferred.

## 1 Introduction

The diagnostic modality of choice for the work up of a thyroid mass is neck ultrasonography. Most patients presenting with chronic thyroid inflammation and a slowly growing goiter causing obstructive symptoms will have a benign cytology. Distinguishing chronic lymphocytic infiltration from a lymphoproliferative process in patients with Hashimoto’s thyroiditis can be particularly challenging with FNAC. We report the case of a 58 y.o. female who presented to an academic center with a slowly growing goiter caused by a thyroid lymphoma not initially diagnosed by FNAC. This case has been reported in line with the SCARE criteria [1].

## 2 Case description

A 58-year-old Caucasian female presented to our clinic with a right thyroid nodule associated to compressive symptoms for 18 months duration, including pressure on her right neck when lying flat and voice changes. She also complained of dry cough while eating and difficulty swallowing solid foods. Unexplained weight loss, fever, diaphoresis, fatigue, anorexia, dyspnea and neck pain were denied.

Pertinent personal medical history included obstructive sleep apnea with nocturnal continuous positive airway pressure use, asthma, and gastroesophageal reflux for the past 3 years. She had no prior history of neck surgery or external radiation. Family history included various types of cancer - pancreatic cancer in her father, lung cancer in her uncle, breast cancer in her mother and sister, and papillary thyroid cancer in her maternal aunt.

A firm mass moving with deglutition was palpated over the right anterior neck on physical exam. There was no tracheal deviation. Voice was clear with full projection, and vocal cords were mobile with normal appearance on flexible laryngoscopy. An elevated TSH (7.36 mIU/L), detectable thyroid peroxidase antibodies (41 IU/mL), and normal free T3 (2.4 pmol/L) and free T4 (1.04 ng/dL) levels were consistent with chronic lymphocytic thyroiditis.

Thyroid ultrasound demonstrated a 5.8 × 2.8 × 3.6 cm right lobe containing two solid hypoechoic, not taller-than-wide thyroid nodules with smooth margins and no echogenic foci, measuring 4.2 cm and 1.8 cm, respectively. Both nodules increased in size during 18-month interval, 1 cm in two dimensions. The left lobe measured 4.3 × 1.4 × 1.3 cm and had no nodules. FNAC was performed at an outside facility (2017), according to the recommendations of the American College of Radiologists for a TIRADS 3 nodule with a growth > 2 mm in two dimensions and over 20% increase in volume. Lymphocytic thyroiditis and absence of malignant cells was reported. A second FNAC prompted by nodule growth revealed similar results 18 months later. Cytology slides review at our institution was concordant.

Cervical and thoracic CT scans were not ordered as the goiter had no substernal extension and there was low suspicion for malignancy on FNAC. Recent esophago-gastroscopy documented no masses or mucosal changes concerning for malignancy.

The patient elected to proceed with a right thyroid lobectomy to relieve her compressive symptoms, after discussing options, including a repeat biopsy. The extent of surgery was planned considering the normal appearance of the left thyroid lobe and absence of nodules on ultrasound, and to minimize her risk for potential surgical complications, including recurrent laryngeal nerve injury and hypoparathyroidism. Surgery was uneventful, she recovered well. Her voice was at baseline and she had no wound complications. Surgical pathology unexpectedly revealed a mixed TL. Overall features were consistent with germinal center subtype DLBCL, rising from a low-grade FL with overexpression of BCL2 and C-MYC. The non-neoplastic areas of thyroid parenchyma were compatible with chronic lymphocytic thyroiditis.

**Fig. 1:**
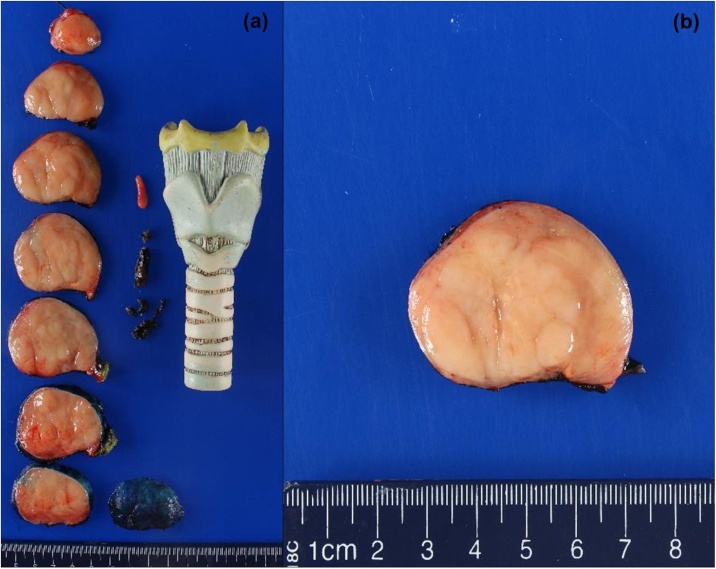
Gross photographs of the lobectomy specimen showing (a) sequential slices from superior to inferior and (b) a closer view of the tan, fleshy, and nodular appearance of the tumor.

**Fig. 2:**
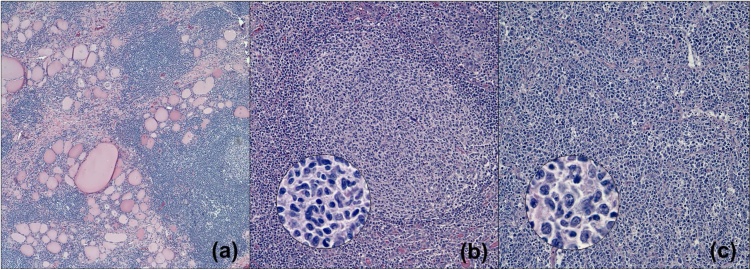
(a) Photomicrograph demonstrating adjacent lymphocytic thyroiditis (hematoxylin and eosin; magnification 100×). (b) Low-power (hematoxylin and eosin; magnification 200×) and high-power (inset; hematoxylin and eosin; magnification 1000× oil immersion) photomicrographs of FL and (c) low-power (hematoxylin and eosin; magnification 200×) and high-power (inset; hematoxylin and eosin; magnification 1000× oil immersion) photomicrographs of DLBCL.

**Fig. 3:**
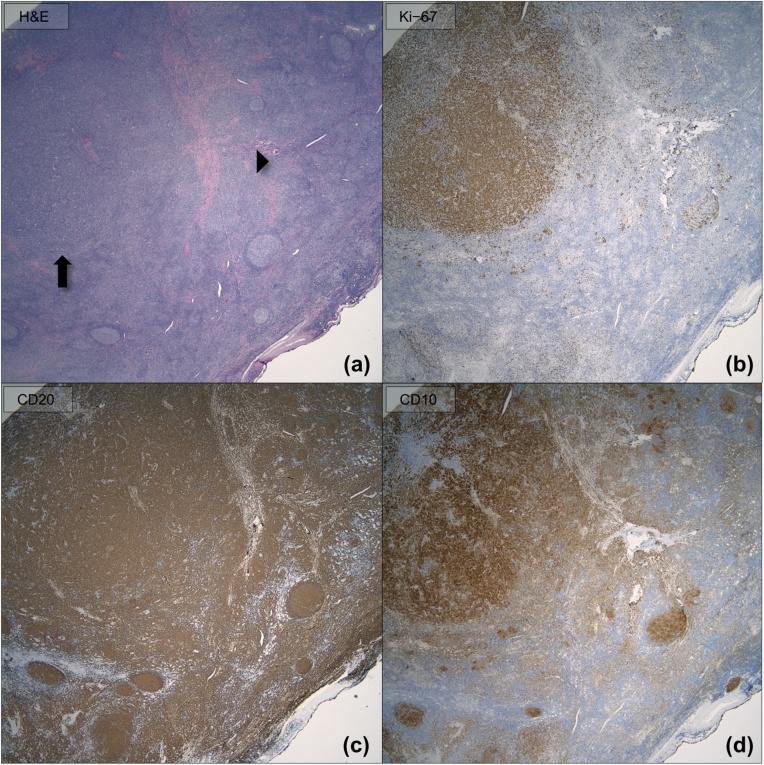
(a) Photomicrograph showing FL (arrowhead) and DLBCL (arrow) replacing normal thyroid parenchyma (hematoxylin and eosin; magnification 200×). (b) Ki-67 proliferation index is approximately 20% in the areas involved by FL and 90% in the areas involved by DLBCL (magnification 200×). The neoplastic B-cells are positive for (c) CD20 (magnification 200×) and (d) CD10 (magnification 200×).

Gross examination (Fig. 1) revealed a 53 g thyroid lobe measuring 6.3 × 5.2 × 4.0 cm, containing a 5.8 cm tumor extending to the inked capsule. Microscopic evaluation (Fig. 2) showed diffuse infiltrates of large pleomorphic cells, and areas with nodular infiltrate of small sized cells with irregular nuclei. The DLBCL infiltrate was estimated at 70% of the tumor while the small cell infiltrate arranged in follicles, composed approximately 30%. The lymphoma did not show extension beyond the boundaries of the thyroid lobe. Immunohistochemistry (IHC, Fig. 3), revealed CD20 + B cells which co-expressed CD10, BCL6 and BCL2, but not cyclin 1. Ki-67 index was 20% in the FL and 90% in the DLBCL component. C-MYC immunostaining was approximately 40% in the DLBCL area. C-MYC gene rearrangement was not identified by interphase fluorescence in-situ hybridization (FISH).

The patient was referred to Oncology for staging and treatment. Bone marrow biopsy showed no tumor involvement. Initial fluodeoxyglucose-18 positron emission tomography (FDG-18 PET) showed an isolated hypermetabolic area in the ipsilateral lower chest and no avid lesions in the abdomen or pelvis, concordant with her cross-sectional imaging. Systemic chemotherapy with rituximab plus cyclophosphamide, doxorubicin, vincristine, and prednisone (R−CHOP) was initiated which she tolerated well, followed by involved-field radiotherapy.

## 3 Discussion

Poorly differentiated thyroid malignancy such as anaplastic cancer, with an annual incidence of 1–2 per million persons, and primary thyroid lymphoma (TL) are part of the differential diagnosis of a rapidly enlarging neck mass with compressive symptoms. Since TL represents only 1–5% of all thyroid malignancies and approximately 2% of all extranodal lymphomas [2,3], there is often a low index of suspicion by the clinician evaluating a thyroid mass. Large population-based studies have identified white, non-Hispanic females in the sixth decade as the predominant patient diagnosed with TL in the United States [4,5].

Hashimoto’s thyroiditis is a well-established risk factor for TL, which may explain the female predominance and later age of presentation when compared to other types of lymphoma. It confers a 67 to 80-fold increased risk in the development of TL and is often a concomitant pathological finding [6,7]. However, in a large Japanese study of 24,553 patients with Hashimoto’s thyroiditis, only 0.5% of the patients developed TL [7].

A rapidly enlarging anterior neck mass in the setting of Hashimoto’s thyroiditis should raise suspicion for TL. Patients usually present with obstructive symptoms of dysphagia, hoarseness, and difficulty breathing. In contrast to other types of lymphoma, TL patients usually do not present with B-type symptoms such as weight loss, night sweats and fever [[4], [5], [6], [7], [8]]. However, the presence of systemic B symptoms at diagnosis increases the risk of mortality.

The sonographic appearance of a solid, hypoechoic thyroid nodule with less echogenicity than that of adjacent thyroid parenchyma and neck muscles is typical of TL. Other characteristics include a variable edge ranging from well to ill-defined, no internal calcifications and increased vascularity, described as a central pattern. Up to 65% of TL lesions are PET avid, with a median SUVmax of 22.7, higher than the median SUVmax 11.1 typical of well-differentiated thyroid cancer [8].

For most thyroid masses, the second diagnostic step undertaken is FNAC. Adequate tissue sampling is essential to grade lymphoproliferative disorders, confirm lymphoma subtype, and conduct IHC studies. While FNAC can diagnose thyroid lymphoma, its sensitivity is inferior to core needle biopsy (CNB) and frequently, additional tissue is required for subtype confirmation. CNB sensitivity has been reported as high as 93%, compared to 71% of FNA in a cohort study at the Mayo Clinic [8]. While the addition of flow cytometry to FNA has increased its sensitivity, there are still diagnostic challenges with this technique related to sample preparation and the amount of tissue available for additional analysis. This case demonstrates that lymphoma should be on the list of differential diagnoses for unexplained thyroid lesions. A negative FNA does not typically rule out the possibility of TL. Hence, core biopsy should be favored in pursuit of complete pathologic characterization. In addition to providing a larger tissue sample for subtype studies like flow cytometry or IHC, CNB provides a specimen with preserved tissue architecture necessary for classification and grading. CNB would also be a more appropriate first diagnostic test in the proper clinical context such as in the case of a rapidly enlarging neck mass where there is a high suspicion for lymphoma. Performing a CNB as a first diagnostic test in a suspected TL can decrease the burden of repeat or more invasive procedures to the patient to establish the correct tissue diagnosis.

TL is a heterogeneous disease presenting with aggressive and indolent histological types. The predominant subtypes in the United States are DLBCL, followed by marginal zone (MZL) and FL respectively. While MZL and FL grow slowly and respond well to treatment, they tend to recur. Transformation of these indolent lymphomas into more aggressive subtypes like DLBCL has been described, and possibly occurred in our patient. DLBCL has the highest grade and the most aggressive course of all TL conferring a worse prognosis, which in addition to older age and advanced disease at presentation (stage IIIE or higher) significantly decreased survival in two national population-based studies [4,5]. While most patients present with stage IE or IIE disease and a median overall survival of 9.3 yr, the 5 yr disease-specific survival rate for DLBCL has been reported as 75%, comparted to 87% for follicular, 86% for lymphocytic, and 96% for MZL [4].

The AJCC Ann Arbor classification defines stage IE (extranodal) as disease confined to the thyroid gland. When the disease has spread to the lymph nodes on the same side of the diaphragm it is classified as stage IIE, as in our patient. Stage IIIE disease involves both sides of the diaphragm and stage IVE has disseminated disease. Appropriate staging workup for TL includes chest, abdomen and pelvis CT scan or MRI, FDG-18 PET scan, and a bone marrow biopsy. Combination chemotherapy plus immunotherapy followed by involved-field radiotherapy is the preferred treatment modality, particularly for disease extending beyond the thyroid gland [[8], [9], [10], [11], [12]]. The R-CHOP regimen is commonly used. For patients with poor response after standard R-CHOP, molecular tumor profiling may show alternative therapy targets. In this setting, sufficient tissue sampling obtained with the least invasive and most efficient technique is of paramount importance.

## 4 Conclusion

TL is a rare malignancy with heterogenous disease progression. While it usually presents as a rapidly growing neck mass, indolent types can present as a slow growing mass with subsequent transformation. When a lymphoproliferative process cannot be excluded by FNAC, performing a CNB helps avoid unnecessary diagnostic steps, including surgery, and potential delays in treatment.

## Declaration of Competing Interest

The authors declare no financial competing interests.

## Funding

This research did not receive any specific grant from funding agencies in the public, commercial, or not-for-profit sectors.

## Ethical approval

This study is exempt from ethical approval at our institution.

## Consent

Written informed consent was obtained from the patient for publication of this case report and accompanying images. A copy of the written consent is available for review by the Editor-in-Chief of this journal on request.

## Author contribution

**Nicole Lin:** Investigation, Writing-Original Draft.

**Susana Vargas-Pinto**: Conceptualization, Writing-Original Draft.

**Courtney E. Gibson**: Writing-Reviewing and Editing.

**Savanah Gisriel:** Investigation, Visualization.

**Mina Xu**: Visualization, Writing-Reviewing and Editing.

## Registration of research studies

1. Name of the registry: N/A*.
2. Unique identifying number or registration ID: N/A*.
3. Hyperlink to your specific registration (must be publicly accessible and will be checked): N/A*.

*Exempt, not a first-man-case report.

## Guarantor

Susana Vargas-Pinto, Courtney E. Gibson.

## Provenance and peer review

Not commissioned, externally peer-reviewed.
